# Enhanced pre-operative thrombolytic status is associated with the incidence of deep venous thrombosis in patients undergoing total knee arthroplasty

**DOI:** 10.1186/1477-9560-12-11

**Published:** 2014-05-27

**Authors:** Yukinori Tamura, Shigeshi Mori, Shigeki Asada, Naoyuki Kawao, Shigeru Ueshima, Hiroshi Kaji, Junichiro Yamamoto, Masao Akagi, Osamu Matsuo

**Affiliations:** 1 Department of Physiology and Regenerative Medicine, Kinki University Faculty of Medicine, 377-2 Ohnohigashi, Osakasayama, Osaka 589-8511, Japan; 2 Department of Orthopaedic Surgery, Kinki University Faculty of Medicine, 377-2 Ohnohigashi, Osakasayama, Osaka 589-8511, Japan; 3 Department of Food Science and Nutrition, Kinki University Faculty of Agriculture, 3327-204 Nakamachi, Nara 631-0052, Japan; 4 Lab oratory of Physiology, Faculty of Nutrition, Kobe Gakuin University, 518 Arise, Igawadani-cho, Nishi-ku, Kobe 651-2180, Japan; 5 Kinki University Faculty of Medicine, 377-2 Ohnohigashi, Osakasayama 589-8511, Japan

**Keywords:** Deep venous thrombosis, Global thrombosis test, Thrombolysis

## Abstract

**Background:**

Deep venous thrombosis (DVT), which is often associated with pulmonary embolism (PE), is a serious complication after total knee arthroplasty (TKA). In the present study, we examined the overall thrombotic and thrombolytic status using Global Thrombosis Test (GTT) in non-anticoagulated blood of patients undergoing TKA to develop the predictable marker for the incidence of DVT.

**Methods:**

DVT was diagnosed using doppler ultrasonography a day after the surgery in 31 patients with osteoarthritis (n = 24), rheumatoid arthritis (n = 6) and ankylosing spondylitis (n = 1) by the well-trained operator. We measured overall thrombotic and thrombolytic status using GTT and other biomarkers, which is associated with blood coagulation and fibrinolysis, before and immediately after the surgery.

**Results:**

Newly-generated DVT during the operation was detected in 11 of 31 patients (35.4%) 1 day after TKA. There were no differences in markers of coagulation (PT and APTT), platelet activity (platelet aggregation-induced by ADP and collagen) and fibrinolysis (FDP and D-dimer) between non-DVT and DVT group both before and after the surgery. Both Pre- and Post-operative GTT-occlusion times (OT), an index of platelet reactivity, were tended to be shorter, but not significant, in DVT group compared with non-DVT group. Pre-operative GTT-lysis time (LT), an index of thrombolytic activity, was significantly shorter in DVT group compared with non-DVT group, while there were no differences in post-operative value of this index between DVT group and non-DVT group, suggesting overall thrombolytic activity was enhanced in DVT group before surgery.

**Conclusions:**

Our data suggest that enhancement of pre-operative thrombolytic activity assessed by GTT may be a predictable marker for the incidence of DVT after TKA.

## Background

Deep venous thrombosis (DVT), which may cause serious pulmonary embolism (PE), is a fatal disease because of its high morbidity and mortality [1-3]. DVT is the most feared complication of major joint arthroplasy such as total hip arthroplasity (THA) and total knee arthroplasty (TKA) [4,5]. Despite advances in surgical technique and clinical managements, patients undergoing these surgeries in the lower limbs remain at high risk for DVT and PE. Although DVT is often occurred within 24–48 hours after TKA, there is also a case that DVT is occurred on the day after this surgery [6]. It is therefore critical to predict the occurrence of DVT as early as possible for the prevention of PE in these patients. However, there is presently no single laboratory marker available to predict or exclusively confirm the diagnosis of DVT [7,8].

The most widely accepted and utilized assay, D-dimer, is highly sensitive and therefore useful for exclusion of the disease but lacks the specificity necessary to confirm the diagnosis [7]. For this reason additional studies including duplex ultrasound, venography, V/Q scanning, helical thoracic and pelvic CT scans and pulmonary angiography remain the standard for diagnosis of DVT and PE.

The global thrombosis test (GTT) is a novel comprehensive test of platelet reactivity, coagulation, and spontaneous thrombolytic activity [9]. As this test is performed from whole (non-anticoagulated) blood, it is genuinely different, and free from many of the shortcomings of conventional platelet test and coagulation, which employ citrate-anti-coagulated blood [9-14]. The aim of the present study was to investigate whether this novel test of overall thrombotic and thrombolytic status can predict the incidence of DVT in patient with undergoing TKA.

## Methods

### Study population

The study protocol was approved by the Ethics Committee of Kinki University Faculty of Medicine (Osakasayama, Japan), and written informed consent was obtained from each patient. The study included 31 patients (3 male and 28 female) with osteoarthritis (n = 24), rheumatoid arthritis (n = 6) and ankylosing spondylitis (n = 1), who underwent TKA consecutively between September 2010 and November 2011 by well-trained operator who has been performing TKA more than 28 years experience of TKA. The patients ranged in age from 55 to 84 years (mean ± SD, 72.3 ± 7.6 years). The medication of anti-coagulant, such as heparin was discontinued a week before surgery in patients undergoing anticoagulative theraphy. The total operation time was 86 ± 11 min.

### Doppler ultrasonography

**Figure 1:**
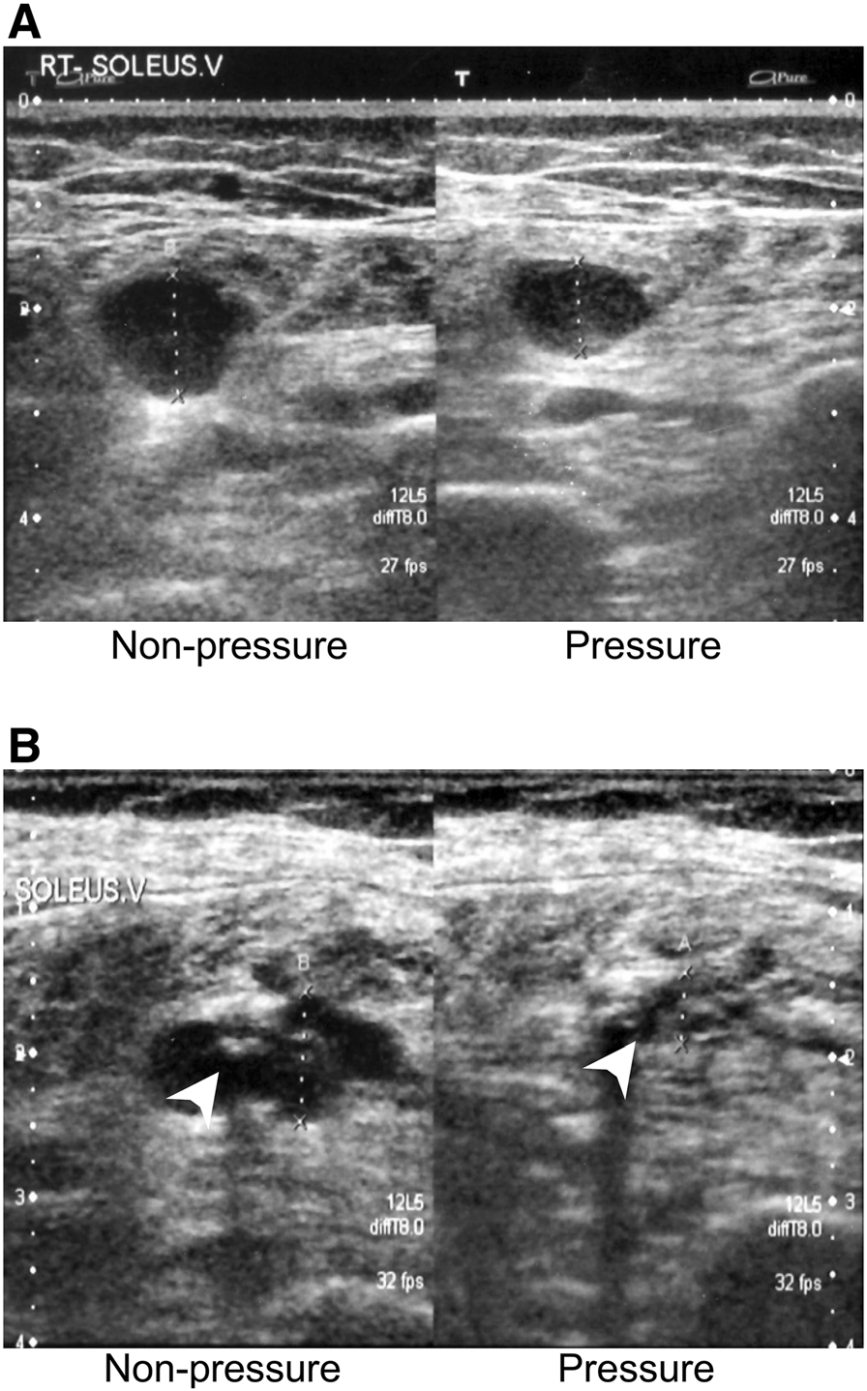
**The difference between newly-generated and aged DVT. A**, **B**: Typical newly-generated **(A)** and aged **(B)** DVT in soleus muscle of patients after TKA with (right panel) or without (left panel) pressure. White arrow indicates the aged venous thrombus in venous of soleus muscle of patients.

Before and a day after TKA, doppler ultrasonography was performed on all patients from the bilateral femoral to lower limb to detect the existence of DVT. Those patients with a previous history of DVT and/or those for which the existence of DVT was detected preoperatively were also included in present study. Aged and newly-generated DVT were distinguished by vessel size and brightness of thrombus. The vessel, in which new thrombus exists, was extended more than 6 mm in diameter, and was not crushed by pressure (Figure 1A). In addition, newly-generated thrombus had low brightness (Figure 1A). In contrast, aged thrombus was small and had high brightness, and the vessel, in which aged thrombus exists, was crushed by pressure (Figure 1B).

### Blood sampling

Blood was collected before and immediately after TKA from antecubital vein using 21 G butterfly needle (TERUMO, Tokyo, Japan) in an air conditioned room (25 ± 0.5°C). The first 11 ml blood was used for measurement of D-dimer, fibrin degradation product (FDP), PT, APTT and for ADP- and collagen-induced platelets aggregation tests, while the subsequent 9.0 ml (for triplicate measurements (3.0 ml in each measurement)) non-coagulated blood was used to perform the Global Thrombosis Test.

### Global thrombosis test (GTT)

**Figure 2:**
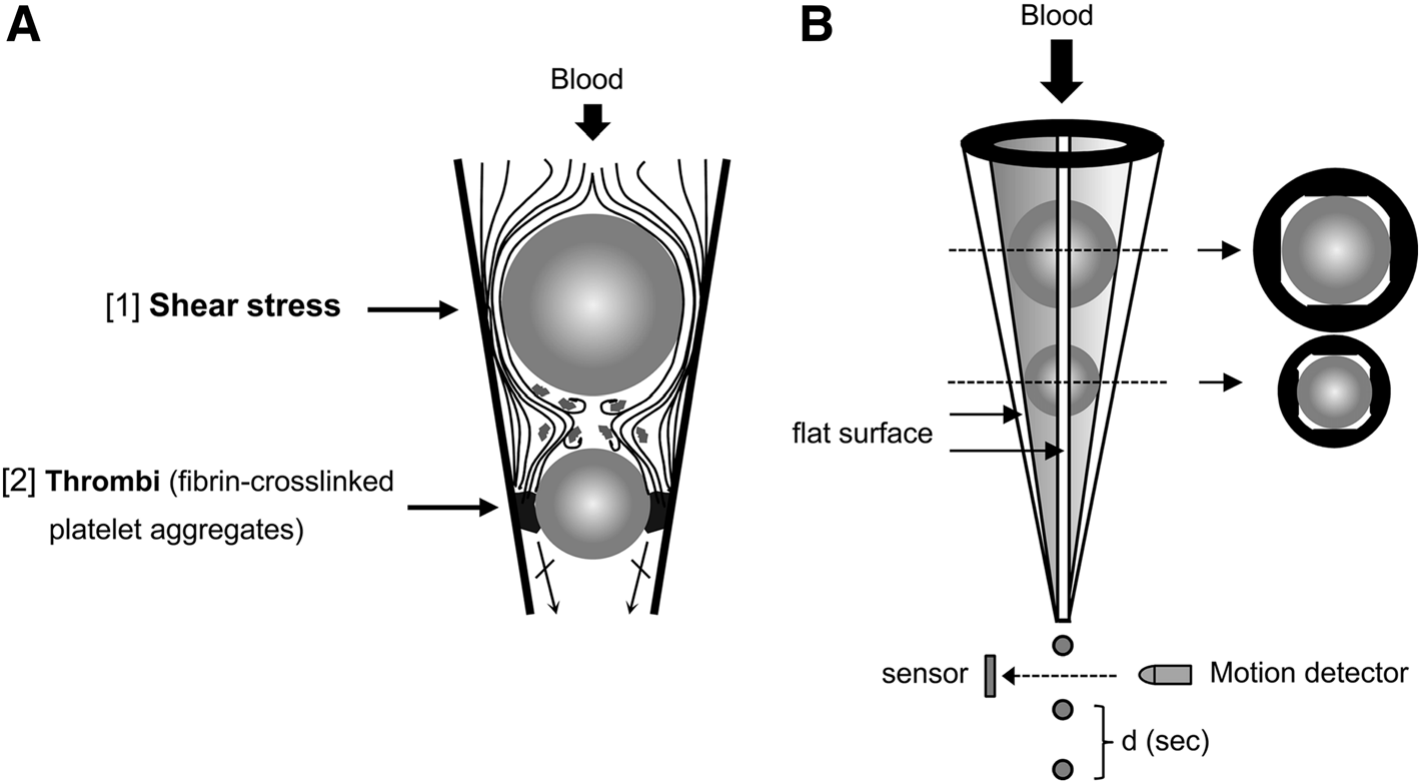
**Principle of the GTT. A**: Blood flows at 37°C under the influence of gravity through a narrow gap [1] formed between the larger ball bearing and the inner wall of the tube, where high shear stress (175 dyn/cm^2^) activates platelets. These activated platelets remain single, since the very short transit time and high shear prevent aggregation. In contrast, in the space downstream, low shear and turbulent flow favour large platelet aggregate formation. The activated platelets generate thrombin and initiate coagulation. The flow then carries these fibrin-stabilized platelet aggregates into the gap resulting in occlusion of the gap and arrest of flow. **B**: A flat segment created along the inner wall of a conical plastic tube forms the basis of the technique, since it prevents the round steel ball bearing from occluding the lumen. When blood is added, it flows through the narrow gaps by the ball and exits in droplets into an adjacent collecting tube. The latter is trans-illuminated and a light sensor generates a signal whenever a drop of blood interrupts the light path. The instrument detects the time interval [d] between consecutive blood drops [2].

The instrument and technique (GTT, Montrose Diagnostics, UK) have previously been described in detail [15]. The principle of the technique is detailed in Figure 2. In brief, non-anticoagulated blood is introduced into, and flows through, a plastic tube in which two metal balls occupy the conical part of the tube. There are four narrow gaps between the inner plastic surface and the balls. When blood flows through the gaps adjacent to the upper ball, the resulting high initial shear stress (175 dyn/cm^2^) causes activation of the platelets. In the space between the balls, due to the turbulent flow and low shear, the activated platelets aggregate. Thrombin is generated, which accelerates the formation of these aggregates and stabilizes them through fibrin. When these stable thrombi reach the gaps around the lower ball, they gradually occlude these gaps, reducing the flow rate and finally stopping the flow of blood. The instrument measures the time (d) between two consecutive blood drops. This time interval increases gradually as flow slows down and at an arbitrary point (d ≥ 15 s, before reaching complete occlusion), the end-point of the measurement is displayed (occlusion time OT; seconds). The restart of blood flow following occlusion is due to spontaneous thrombolysis (lysis time, LT; seconds). If lysis does not occur until 6000 s following OT (LT cut-off time), “no thrombolysis” is recorded. We averaged the data from triplicate measurement of GTT.

### Statistical analysis

The values of OT and LT were shown as the medians (minimum and max values), and comparisons between groups are evaluated using non-parametric methods (Mann–Whitney U test). Other parameters were expressed as means ± SD, and statistical significance in independent pairs (non-DVT and DVT group) and dependent groups (before and after surgery) were analyzed by unpaired and paired t test, respectively. P value less than 0.05 was considered as limit of significance. These calculations were made with the software package Stat View (ver. 5.0, SAS Institute, NC, USA). Analysis based on receiver-operating characteristic (ROC) curve was performed with the SPSS software package (ver. 18, International Business Machines Corporation, NY, USA).

## Results

### The prevalence of DVT and the characteristic of non-DVT and DVT group

DVT was diagnosed in 11 of 31 patients resulting in an incidence of 35.4% (Table 1). There was no pulmonary embolism. Although DVT group had lower body weight compared with DVT group, no significance was observed in age and BMI between non-DVT group and DVT group (Table 1). The aged DVT was detected pre-operatively in 54.5% of patient with post-operative incidence of DVT before surgery (Table 1).

**Table 1:** Characteristic of non-DVT and DVT groups

	**Non-DVT (n = 20)**	**DVT (n = 11)**	**p value**
Male/female	1/19	2/9	
Age (year)	70.7 ± 8.5	75.1 ± 5.2	0.127
Height (cm)	153.0 ± 6.8	150.6 ± 6.2	0.249
Weight (kg)	60.3 ± 10.5	52.9 ± 6.9	0.046
BMI (kg/m^2^)	25.4 ± 3.7	23.6 ± 3.2	0.196
Diagnosis (number (%))			
Osteoarthritis	15 (75.0%)	9 (81.8%)	
Rheumatoid arthritis	5 (25.0%)	1 (9.1%)	
Ankylosing spondylitis	0 (0%)	1 (9.1%)	
Medical history (number (%))			
Aged DVT	1 (5.0%)	6 (54.5%)	
Hypertension	15 (75.0%)	7 (63.6%)	
Diabetes mellitus	2 (10.0%)	3 (27.2%)	
Hyperlipidemia	5 (25.0%)	3 (27.2%)	
CVD	3 (15.0%)	0 (0%)	
Stroke	0 (0%)	1 (9.1%)	
Cancer	1 (5.0%)	0 (0%)	
Hyperuricemia	3 (15.0%)	0 (0%)	

CVD; cardiovascular disease.

### Parameters of blood coagulation and platelet activity

The values of PT (sec) was slightly increased in DVT group immediately after the surgery compared with before surgery, while there was no difference in the values of PT and APTT between non-DVT and DVT group both before and immediately after surgery (Table 2). Platelets number and platelet aggregation-induced by ADP or collagen were also not observed significant difference between non-DVT and DVT group both before and immediately after surgery (Table 2).

**Table 2:** Parameters of coagulation, aggregation and fibrinolysis in non-DVT and DVT groups before or after TKA

	**Before TKA**			**After TKA**		
	**Non-DVT**	**DVT**	**p value**	**Non-DVT**	**DVT**	**p value**
PT (sec)	11.4 ± 0.7	11.4 ± 0.6	0.938	11.9 ± 0.6	12.3 ± 0.7†	0.088
PT (%)	99.2 ± 14.6	98.0 ± 11.8	0.825	87.0 ± 21.8*	82.9 ± 10.3†	0.568
PT (INR)	1.01 ± 0.07	1.01 ± 0.05	0.984	1.04 ± 0.07	1.09 ± 0.06†	0.078
APTT (Sec)	27.2 ± 3.0	26.9 ± 1.8	0.793	28.1 ± 3.6	28.2 ± 2.1	0.926
Platelets (10^4^/μl)	24.2 ± 7.9	23.3 ± 6.2	0.750	22.4 ± 7.5	21.4 ± 5.5	0.686
ADP2.5 (%)	56.1 ± 16.5	53.0 ± 16.7	0.645	52.6 ± 17.1	50.5 ± 13.6	0.746
ADP5.0 (%)	71.2 ± 14.6	69.2 ± 17.3	0.746	65.3 ± 13.2	68.3 ± 13.9	0.575
ADP10.0 (%)	81.3 ± 17.5	79.7 ± 14.4	0.810	74.8 ± 16.3	73.9 ± 29.2	0.919
Col1.0 (%)	47.8 ± 30.4	49.0 ± 23.0	0.905	35.1 ± 29.1	25.3 ± 25.9	0.384
FDP (μg/ml)	4.7 ± 2.0	4.4 ± 1.3	0.671	5.3 ± 2.5	5.2 ± 2.3	0.953
D-dimer (μg/ml)	1.3 ± 1.0	1.0 ± 0.5	0.301	2.2 ± 1.8*	3.0 ± 2.5†	0.311

ADP2.5, ADP5.0, ADP10.0; Platelet aggregation-induced by ADP (2.5, 5.0 and 10.0 μg/ml, respectively). Col1.0; platelet aggregation-induced by collagen (1.0 μg/ml). *; p < 0.05 vs non-DVT group before surgery, †; p < 0.05 vs DVT group before surgery.

### Parameter of fibrinolysis

No differences were observed in the values of FDP, a marker of primary and secondary fibrinolysis, between non-DVT and DVT patient both before and immediately after surgery (Table 2). The post-operative values of D-dimer, a marker of secondary fibrinolysis, was increased both in non-DVT group and DVT group, while there was no difference in the values of D-dimer between non-DVT and DVT group both before and immediately after surgery (Table 2).

### Overall thrombotic and thrombolytic status assessed by GTT

To assess the overall thrombotic and thrombolytic status, GTT was performed in patients before and immediately after the surgery. The pre- and post-operative values of OT, which reflects the thrombotic activity, were tended to be shorter in DVT group compared with non-DVT group (Table 3), while there was no significant difference in these values of OT between non-DVT group and DVT group (Table 3). The pre-operative values of LT, an index of the endogenous thrombolytic activity, in DVT group was markedly shorter than that in non-DVT group, whereas no difference was observed in the post-operative values of LT between DVT and non-DVT groups (Table 3). These data suggest that the pre-operative endogenous thrombolytic activity is enhanced in DVT group.

**Table 3:** OT and LT in non-DVT and DVT groups before or after TKA

	**Before TKA**			**After TKA**		
	**Non-DVT**	**DVT**	**p value**	**Non-DVT**	**DVT**	**p value**
Median OT (Sec)	460.8	417.1	0.069	498.5	381.1	0.083
(Min)	314.2	313.9		249.0	307.7	
(Max)	665.1	531.9		778.2	539.8	
Median LT (Sec)	2962.0	1852.7	0.003	2560.5	2880.5	0.804
(Min)	1679.0	1383.0		1366.9	1670.0	
(Max)	5222.0	2992.5		5952.0	3905.0	

### The sensitivity and specificity of LT before TKA in DVT

The calculations of sensitivity and specificity of LT before TKA in the detection of DVT are shown in Table 4. When LT (<2600 sec) before the surgery is used as a marker of DVT immediately after the surgery, sensitivity is 90.9%, specificity is 65.0%. We also performed the analysis based on receiver-operating characteristic (ROC) curve using statistical software. This analysis revealed that highest combined sensitivity (90.9%) and specificity (65.0%) were shown when cut-off value was set 2593 sec (LT before the surgery).

**Table 4:** Sensitivity and specificity of different preoperative LT in DVT after TKA

	**DVT (n = 11)**	**Non-DVT (n = 20)**	**Sensitivity (%)**	**Specificity (%)**
LT (sec)				
<3000 vs >3000	11 vs 0	10 vs 10	100.0	50.0
<2800 vs >2800	10 vs 1	9 vs 11	90.9	55.0
<2600 vs >2600	10 vs 1	7 vs 13	90.9	65.0
<2400 vs >2400	9 vs 2	7 vs 13	81.8	65.0
<2200 vs >2200	7 vs 4	6 vs 14	63.6	70.0
<2000 vs >2000	6 vs 5	5 vs 15	54.5	75.0

## Discussion

It is critical to predict the incidence of DVT after major joint arthroplasity such as TKA and THA because of high frequency (more than 40%) in the incidence of DVT after the surgery [4,5]. The formation of thrombosis is multifactorial. Virchow's triad (hypercoagulability, endothelial injury, and venous stasis) established the basis of understanding the pathogenesis of venous thrombosis [16-18]. Therefore, clinical method, which can assess the thromobotic and thrombolytic status more physiologically, has been necessary than single biomarker to predict the incidence of DVT. However, many clinical researchers have made an attempt to predict or diagnose the DVT using single biomarker, or agonist-induced platelet aggregation, due to the technical limitation [7].

It has been suggested that the D-dimer test may be useful in early detection of DVT. Many studies of non-traumatized patients reported that the plasma D-dimer assay is indicative of DVT [19-21]. However, the value of D-dimer in DVT after TKA remains controversial. Some report showed that a high level of D-dimer was most sensitive with 94.4% for TKA and most specific with 90.0% for TKA in the diagnosis of DVT after TKA [22]. In contrast, other studies reported a negative value of D-dimer in the diagnosis of DVT after TKA. Bounameaux et al. reported that measurement of plasma D-dimer concentration is not value for predicting, diagnosing or ruling out DVT in patient undergoing TKA [23]. Harper et al. also stated that the D-dimer measurement is too insensitive to use as reliable exclusion test in cases of suspect [24]. We also observed no difference in D-dimer between DVT and non-DVT patients both before and after TKA, whereas D-dimer levels were increased after TKA in both non-DVT and DVT patients. An elevated D-dimer does not always indicate the presence of a clot because a number of other factors can cause an increased level. It has been suggested that elevated levels may be seen in conditions in which fibrin is formed and then broken down, such as surgery, trauma, infection, heart disease, and some cancers or conditions in which fibrin is not cleared normally, such as liver disease [25]. Therefore, D-dimer test alone may be not accurate enough in prediction or detection of early DVT. This limitation of D-dimer test may reflect the limitation for predicting the DVT using single serum biomarker.

In the present study, we assessed the feasibility of GTT as a novel marker for predicting the incidence of DVT after TKA as a pilot study, which has been operated by the well-trained operator. This design could avoid the influence to the result due to the operator’s technique. The unique features of GTT are employing non-anticoagulated blood and solely high shear to trigger platelet activation, which makes this more physiological than other platelet function tests performed on citrate-anticoagulated blood or those employing various exogenous agonists to stimulate platelets [10]. In the GTT, initial activation of platelets is due to high shear stress, akin to that in an artery with a 70% luminal stenosis, on which thrombosis is likely to occur [26]. Subsequently ADP released from shear-activated platelets and red cells [27] aggregates platelets and induces thromboxane formation. Activated platelets generate thrombin, accelerating aggregation and fibrin stabilizes the labile platelet aggregates. Thus, this technique takes into account all the major players in thrombus formation (high shear, ADP, and thrombin) and is the closest approximation to thrombotic formation in vivo, and can detect overall thrombotic status.

A further advantage of the GTT is that it allows for the first time the measurement of endogenous thrombolytic activity [28]. Due to the previous lack of a global fibrinolysis test, a battery of markers of the activators and inhibitors of the fibrinolytic system have been used but the practical value of these fibrinolysis markers is low and questionable [29]. Thrombolysis, as opposed to clot lysis, could not be measured. The GTT is the unique technique to measure formation and then lysis of platelet-rich thrombi, providing a true assessment of endogenous thrombolytic status.

In the present study, we found that pre-operative LT in DVT patients was shorter than that in non-DVT patients. Furthermore, the present study showed that pre-operative LT of shorter than 2600 sec is sensitive and moderator specific enough in the prediction of DVT after surgery. Thrombosis is induced by the alteration of blood components, blood flow and blood vessel structure [16]. The vessel occlusion due to the compression of the leg with high pressure during the operation and re-perfusion of blood flow after the operation might be associated with the occurrence of DVT at the local site of vessel in the leg after TKA. OT is the time to form an occlusive thrombus under high shear stress in the GTT plastic tube. LT is the time of the restart of blood flow following occlusion in the GTT plastic tube, and may be affected by the fibrinolytic activity, shear stress and the degree of aggregation of thrombus generated in the GTT plastic tube. Therefore, pre-operative LT assessed in systemic venous blood sample might not simply reflect the fibrinolytic status, and might be also not directly linked with thrombotic status of the local site where DVT may occur. In contrast to pre-operative LT, post-operative LT were not observed significant differences between DVT and non-DVT patients. This might be due to the elevation of a lot of factor, such as coagulation, anti-coagulation, fibrinolysis factor, and inhibitor of fibrinolysis during and at the end of total joint arthroplasty [30]. We also observed pre- and post-operative OT were not significant difference between non-DVT and DVT group, but these values were tended to be shorter in DVT group compared with non-DVT group. Taken together, pre-operative LT may be a novel marker for predicting the occurrence of DVT after TKA. However, further study including larger population would be necessary to establish GTT as predictable tool of DVT.

## Conclusions

We showed the possibility of pre-operative endogenous thrombolytic status assessed by GTT as a predictable marker for the occurrence of DVT after TKA. Because GTT can be used at bedside, and it is very easy to apply, GTT may be a beneficial tool for prediction of DVT in patients undergoing total joint replacement therapy.

## Abbreviations

DVT: Deep venous thrombosis; THA: Total hip arthroplasity; TKA: Total knee arthroplasty; GTT: Global thrombosis test; FDP: Fibrin degradation product; OT: Occlusion time; LT: Lysis time.

## Competing interests

The authors declare that they have no competing interests.

## Authors’ contributions

YT participated in all experiments, drafted manuscript and performed the statistical analysis. SM, SA and AM participated in all experiments and helped to draft the manuscript. NK, US, HK, and JY helped to draft the manuscript. OM conceived of the study, and participated in its design and coordination and drafted manuscript. All authors read and approved the final manuscript.
